# Comparison of carbon footprint and net ecosystem carbon budget under organic material retention combined with reduced mineral fertilizer

**DOI:** 10.1186/s13021-021-00170-x

**Published:** 2021-03-01

**Authors:** Ying Liu, Haiying Tang, Pete Smith, Chuan Zhong, Guoqin Huang

**Affiliations:** 1 College of Agriculture and Biotechnology, Hunan University of Humanities, Science and Technology, Dixing Road, Louxing District, Loudi, 417000 Hunan China; 2 Research Center on Ecological Sciences, Jiangxi Agricultural University, No. 1101 Zhimin Avenue, Nanchang Economic and Technological Development Zone, Nanchang, 330045 Jiangxi China; 3 Institute of Biological and Environmental Sciences, School of Biological Sciences, University of Aberdeen, 23 St Machar Drive, Room G45, Aberdeen, AB24 3UU Scotland, UK; 4 College of Agriculture, Nanjing Agricultural University, Nanjing, 210095 China

**Keywords:** *Astragalus sinicus* L., Rice straw, Reduced mineral fertilizer, Carbon footprint, Greenhouse gas emissions, Net ecosystem carbon budget

## Abstract

**Background:**

Excessive application of chemical fertilizer has resulted in lower nitrogen uptake and utilization efficiency of crops, decreasing soil fertility, increasing greenhouse gas emissions, and worse environmental pollution. Organic material retention is regard as the key to solve these problems. The objective of this study is to conduct an assessment of carbon budget under *Astragalus sinicus* L. and rice straw retention combined with reduced mineral fertilizer based on the 2-year field experiment in a paddy field in the south of China. The experiment was randomized complete block design including four treatments with triplicates: control CK (winter follow, 120 kg ha^−1^ N fertilizer for each rice season) and three treatments with *Astragalus sinicus* L. and rice straw retention named RA, RB, and RC (reduced N fertilizer by 15%, 27.5%, and 40% in each rice season).

**Results:**

Treatments RA, RB, and RC increased greenhouse gas emissions by 9.30–101.25%, among which CH_4_ accounted for more than 60%; Carbon input of crops from treatments RA, RB, and RC increased by 2.25–12.10% compared with control CK over the 2 years. Though treatments RA, RB, and RC enhanced CO_2_ emissions, treatment RB decreased carbon footprint and became carbon sink.

**Conclusions:**

The results of this study reveal that treatment RB (*Astragalus sinicus* L. and rice straw retention with reduced N fertilizer by 27.5%) is better in reducing chemical fertilizer amount, increasing crop yield and carbon input, which is more conductive to sustainable development of agriculture.

**Supplementary Information:**

The online version contains supplementary material available at 10.1186/s13021-021-00170-x.

## Background

Carbon (C) footprint refers to the total carbon dioxide (CO_2_) emissions generated directly or indirectly by an activity or product throughout its life cycle and expressed in CO_2_ equivalent (CO_2_-eq) [1]. Greenhouse gas (GHG) emissions from agriculture accounts for 20–30% in the globe [2]. The C footprint in agriculture can systematically evaluate the indirect C emissions (diesel, electricity, fertilizer, pesticide and agricultural film) from agricultural inputs and the total amount of direct C emissions [3]. The C budget and balance includes C input (mostly coming from crop C sequestration) and C output (direct and indirect GHG emissions) in agriculture ecosystem.

Rice is one of the important crops in the world while paddy field is also an important agriculture GHG emissions source [4]. Rice planting area in China occupies approximately 19% in the world [5]. With the increase of population in the future, the demand for rice will inevitably increase, which will consume more energy, chemical fertilizers and pesticides, contributing directly and indirectly to GHG emissions from farmland. As an important greenhouse gas, CO_2_ contributes 60% to global warming, of which about 5–20% comes from farmland soil [6]. According to the fifth report of IPCC, the atmospheric concentrations of CO_2_ had reached 391 ppm by 2011, which were 40% higher than that before the Industrial Revolution [7]. Methane (CH_4_) and nitrous oxide (N_2_O) emissions from paddy fields in China account for 17.9% and 80% of the total emissions and their concentrations are also increasing at the speed of 0.03 and 0.75 ppb year^−1^ in recent years [8–10].

Meanwhile, farmland ecosystem is also an important system for C sequestration and GHG mitigation. Increasing studies indicate that straw retention can sequestrate C and mitigate GHG emissions through directly inputting soil organic carbon (SOC) and increasing C storage [11, 12]. China is abundant with crop straw resources, with an average annual production of 7.6–8.2 million tons [13], accounting for about 25% in the world [14] and the rice straw in the south of China accounts for about 50–60% [15].

Winter green manure and double-rice rotation is a traditional planting pattern in the south of China. *Astragalus sinicus* L. and rice straw contain a lot of nutrients and their reasonable application can not only replace part of chemical fertilizer, solve the adverse problems caused by excessive application of chemical fertilizer [16], but also avoid the waste of resources and environmental pollution resulted from straw burning [17] as well as increase SOC content [11, 12]. However, increased CH_4_ emissions in paddy field after straw retention may offset GHG emissions mitigation effect of soil C sequestration [18, 19], which can not be ignored as an important GHG leakage. To clarify whether the reduced mineral fertilizer under *Astragalus sinicus* L. and rice straw retention can lower GHG emissions and enhance C sink, it is necessary to conduct an analysis to reveal whether there are trade-offs between these two indicators by using C footprint and net ecosystem carbon budget (NECB).

At present, most studies mainly focus on the effect of different tillage systems and different rotation patterns on C footprint [20–22]. Some researchers use the available data to calculate C footprint or use remote sensing and numeric modeling to investigate the water–carbon interactions or simulate C sequestration [23–27]. However, little is known on comprehensive effects of reduced mineral fertilizer under organic material retention on C footprint and NECB. To provide theoretical basis for C sequestration and emissions mitigation of paddy field and sustainable development of agriculture, we conducted a 2-year field experiment to test the following hypotheses: (1) whether organic material retention combined with reduced mineral fertilizer can increase crop C input? (2) whether C input can offset the increased GHG emissions? (3) Whether fertilizer and year had interactive effect on C footprint and NECB?

## Methods

### Experiment site characteristics

The field experiment was conducted in Yujiang County, Yingtan City from 2017 to 2019. This place belongs to subtropical monsoon humid climate with mean annual temperature and precipitation of 17.6 °C and 1741 mm, respectively. Most of the soils are silt-deposited soils and a few are red loam soils. Before the experiment, the pH, the content of organic matter, total nitrogen, total phosphorus, and total potassium in surface soil (0–15 cm) were 5.12, 34.7 g kg^−1^, 1.9 g kg^−1^, 0.66 g kg^−1^, and 15.33 g kg^−1^.

### Experiment design and management

The experiment adopts split plot design. The main zone includes two kinds of rice straw retention amount (0 and 6000 kg ha^−1^). The secondary zone includes reduced chemical fertilizer at three different rates compared with control CK. There are four treatments with triplicates (Table 1): CK (winter fallow, without organic materials retention and 120 kg ha^−1^ N fertilizer was applied for each rice season), and three treatments with *Astragalus sinicus* L. and rice straw retention combined with reduced mineral fertilizer named RA (− 15% N fertilizer for each rice season), RB (− 27.5% N fertilizer for each rice season), and RC (− 40% N fertilizer for each rice season). Each plot area is 25 m^2^ (5 m × 5 m), around which there are protection lines to prevent water and fertilizer cross-contamination.

**Table 1:** Field experimental design

Treatments	Chinese milk vetch retention amount (kg ha^−1^)	Rice straw retention amount (kg ha^−1^)	N application of each rice season (kg ha^−1^)
CK	0	0	120
RA	Full	6000	− 15%
RB	Full	6000	− 27.5%
RC	Full	6000	− 40%

The pure phosphorus and potassium was 20 kg ha^−1^ and 60 kg ha^−1^ respectively. 60%, 30%, and 10% N fertilizer (N 46%) were used as basic, tiller and panicle fertilizer respectively. Phosphorus fertilizer (P_2_O_5_ 12%) was used as basic fertilizer and applied once. 70% and 30% potassium fertilizer (K_2_O 60%) was applied as tiller and panicle fertilizer. The N and P basic fertilizers were applied 1 day before rice transplanting, the tiller fertilizer was applied 5–7 days after rice transplanting and the panicle fertilizer was applied when the main stem was 1–2 cm long.

### Experiment materials

The variety of *Astragalus sinicus* L. was Yujiang Daye. Seeds of 37.5 kg ha^−1^ were sown on 3 October in 2017 and 7 October in 2018, and they were weighted, mixed, calculated the average value (retention amount of *Astragalus sinicus* L. was the same for each plot except control CK), and plowed into the field at the blooming stage in the middle of April of next year. The early rice was “Yueru No. 6”, which was transplanted on 26 April 2018 and 25 April 2019 and harvested on 12 July 2018 and 11 July 2019; the late rice was “Huarun No. 2”, which was transplanted on 18 July 2018 and 15 July 2019 and harvested on 2 November 2018 and 16 November 2019. After the early rice harvest, the straw was cut into 3–5 cm sections with a guillotine, and then plowed into the field. After the late rice harvest, the straw was left and covered the field. The residue height of rice was 2–3 cm.

### Measurement of items and methods

#### Collection and measurement of GHG

GHG were collected by using static chamber with the size of 50 cm × 50 cm × 50 cm. When the rice plant exceeded 50 cm, the other chamber with the same size and two-way opening was added. There is one fixed sampling base with a groove of 5 cm depth filled with water when collecting the gas samples at per plot. Samples were collected from 8:00 to 11:00 every 7–8 days during rice growth period and every 15 days [28] in *Astragalus sinicus* L. growth season, respectively. A 50 ml syringe was used to extract the gas at 0, 10, 20 and 30 min and the syringe was pulsed back and forth 5–10 times to evenly mix the gas. After the gas was extracted and stored in vacuum bags, gas samples were quickly taken back and analyzed by using Agilent 7890A gas chromatography.

#### Calculation of GHG

The GHG flux is calculated according the equation:

1 $${\text{F}} = \rho \times {\text{h}} \times {\text{dc}}/{\text{dt}} \times 273/\left( {273 + {\text{T}}} \right)$$

where F is the gas emissions flux, ρ is the gas density under standard conditions (kg m^−3^), h is the net height (m) of sampling chamber, dc/dt is the change rate of gas concentration in the sampling chamber per unit time, T is the average temperature (°C) in the sampling chamber during sampling process, and 273 is the constant of the gas equation.

The cumulative emissions of CH_4_ and N_2_O from paddy fields were calculated as follows:

2 $${\text{Tn}} = \sum\limits_{i = 1}^{{\text{n}}} {F_{{\text{i}}} } *{\text{D}}_{{\text{i}}}$$

where Tn is annual cumulative emissions, F_i_ is the average daily emissions flux of CH_4_ and N_2_O between two sampling periods; D_i_ is the number of days between two sampling intervals.

#### C footprint calculation

According to PAS 2050 [29], C footprint of agricultural production is calculated as the sum of all indirect and direct GHG emissions during one crop production in a certain cropping system (kg CO_2_-eq ha^−1^) based on life cycle assessment and expressed in CO_2_ equivalent (CO_2_-eq). Therefore, in this study, C footprint of *Astragalus sinicus* L. and rice production includes indirect and direct GHG emissions, of which the former are from agricultural inputs (fertilizers, pesticides, machinery, electric irrigation) while the latter are from CH_4_ and N_2_O emission in paddy field. GHG emissions from agricultural inputs are estimated using the following formula:

3 $${\text{CE}}_{{{\text{input}}}} = \sum \left( {{\text{A}}_{{\text{i}}} \times \delta_{{\text{i}}} } \right).$$

In the formula, CE_input_ refers to the total GHG emissions (kg CO_2_-eq ha^−1^) from agricultural inputs, *i* refers to a certain agricultural input, Ai is the intensity or quantity of the *i*th individual agricultural input (pesticide/fertilizer, kg ha^−1^; electricity, kwh ha^−1^; Diesel, L ha^−1^), and δ_i_ is the coefficient factors of the *i*th individual agricultural input. The GHG emissions factors from agricultural inputs are shown in Table 2.

4 $${\text{CF}} = \left( {{\text{CE}}_{{{\text{input}}}} + {\text{EN}}_{{2}} {\text{O}} + {\text{ECH}}_{{4}} } \right)/{\text{Y}}$$

**Table 2:** Agricultural inputs (Ai), and related coefficient factors (δ_i_) and application rate

Treatments	GHG emission source from agricultural inputs	Emission coefficient	Agricultural inputs			
			Unit	Application rate		
				Chinese milk vetch	Early rice	Late rice
CK	N fertilizer	6.38	kg ha^−1^	0	120	120
RA	N fertilizer	6.38	kg ha^−1^	0	102	102
RB	N fertilizer	6.38	kg ha^−1^	15	87	87
RC	N fertilizer	6.38	kg ha^−1^	30	72	72
Same for all the treatments	P fertilizer	0.44	kg ha^−1^	0	20	20
Same for all the treatments	K fertilizer	0.61	kg ha^−1^	0	60	60
Same for all the treatments	Diesel for machinery	2.63	kg ha^−1^	41	70	70
Same for all the treatments	Pesticide	14.0	kg ha^−1^	7	13	13
Same for all the treatments	Electricity for irrigation	1.12	Kg ha^−1^	0	468	468

The data were obtained from the average value of agricultural input in this study. N represents nitrogen fertilizer; P represents phosphate fertilizer; K represents potash fertilizer; GHG represents greenhouse gas

In the formula, CF refers to C footprint; ECH_4_ and EN_2_O refers to CH_4_ and N_2_O cumulative emissions, which are converted to CO_2_-eq from soils during *Astragalus sinicus* L. and rice growth season; Y refers to the biomass of *Astragalus sinicus* L. and rice yield (kg ha^−1^).

#### Total C input and NECB

Total C input based on C sequestration in biomass was estimated using the following equation [30].

5 $${\text{E}}_{{{\text{input}}}} = {\text{B}}_{{{\text{total}}}} \left( {{\text{B}}_{{{\text{grain}}}} + {\text{B}}_{{{\text{straw}}}} + {\text{B}}_{{{\text{root}}}} + {\text{B}}_{{{\text{litter}}}} + {\text{B}}_{{{\text{rhizodeposites}}}} } \right) \times {\text{f}}_{{\text{c}}} \times \left( {{44}/{12}} \right)$$

Crop yield and straw were weighed on site; root biomass, litter, and rhizodeposits are calculated according to Salam et al. [31] and Huang et al. [32]; fc is the C percentage in grain (40% for rice) [33].

6 $$\begin{aligned} {\text{NECB}} = \,& {\text{E}}_{{{\text{input}}}} - {\text{E}}_{{{\text{output}}}} \left({\text{CO}}_{{2}} {\kern 1pt} {\text{equivalent of CH}}_{{4}} \;{\text{and N}}_{{2}} {\text{O cumulative emissions plus}}\, {\text{CO}}_{{2}} \;\right. \\ & \left. {\text{emissions from plant respiration and soil microbial respiration}} \right). \end{aligned}$$

### Data analysis

A statistical analysis was performed using Microsoft Excel 2010 and SPSS 17.0. Origin 9.0 was used to create a diagram. A mixed linear model was used to analyze the effects of fertilizer and year on mean GHG, CO_2_, C input, C footprint, crop biomass, and NECB during the crop growing season. Mean values for each variable were compared by a one-way ANOVA, followed by a Duncan’s post hoc test (*P* < 0.05).

## Results and discussion

### GHG emissions

The GHG emissions from all the treatments include indirect emissions from agricultural inputs (Table 2) and direct CH_4_ and N_2_O emissions (Table 3), among which the former accounts for more than 17% and the latter occupies more than 60%. The GHG emissions from all the treatments ranged from 9731 to 19,584 kg CO_2_-eq ha^−1^ and treatments RA, RB and RC with organic materials retention combined with reduced mineral fertilizer increased by 9.30–101.25% compared with that of control CK over the 2 years. The difference of GHG emissions between treatments RA, RC and control CK was significant (*P* < 0.05), while the difference between treatment RB and control CK was insignificant (Table 3), which may be caused by the different turnover depth and decomposition rate of *Astragalus sinicus* L. and rice straw in each plot. The study result of Zhu et al. [34] indicated that different depth of straw retention (0–10 cm, 10–20 cm, 20–30 cm, 30–40 cm) had different effects on GHG emissions. The reason may be that the different depth of straw retention made the straw lie in different soil layers with different natural conditions and microbial diversity, which affected straw decomposition rate [35, 36] and SOC content [37], thus affecting GHG emissions. From Table 5 we can see that straw retention had significant effect on GHG, C input, and crop biomass. Year had significant impact on CO_2_ and NECB. Moreover, fertilizer and year had significant effect or interactive effect on GHG emissions, CO_2_, C footprint, and NECB.

**Table 3:** Average annual GHG emissions and C footprint during crop growth seasons over the two years (kg CO_2-_eq ha^−1^)

T	Indirect emission						Direct emission		Average GHG emissions	Yield ( kg ha^−1^)	Carbon footprint ( kg CO_2_-eq kg^−1^ grain)
	N	P	K	Diesel	Electricity	Pesticides	CH_4_	N_2_O			
CK	1531	18	73	476	1048	462	5863c	260	9731c	15209b	0.63c
RA	1301	18	73	476	1048	462	16037a	169	19584a	19479a	1.01a
RB	1206	18	73	476	1048	462	7164c	189	10636c	20530a	0.52c
RC	1110	18	73	476	1048	462	13577b	261	17025b	20124a	0.85b

T represents treatment; GHG represents greenhouse gas; C represents carbon; yield represents Chinese milk vetch straw and rice biomass. The different lowercase letters indicate significant differences among treatments at *P* < 0.05

### C footprint components of all the treatments

**Fig. 1:**
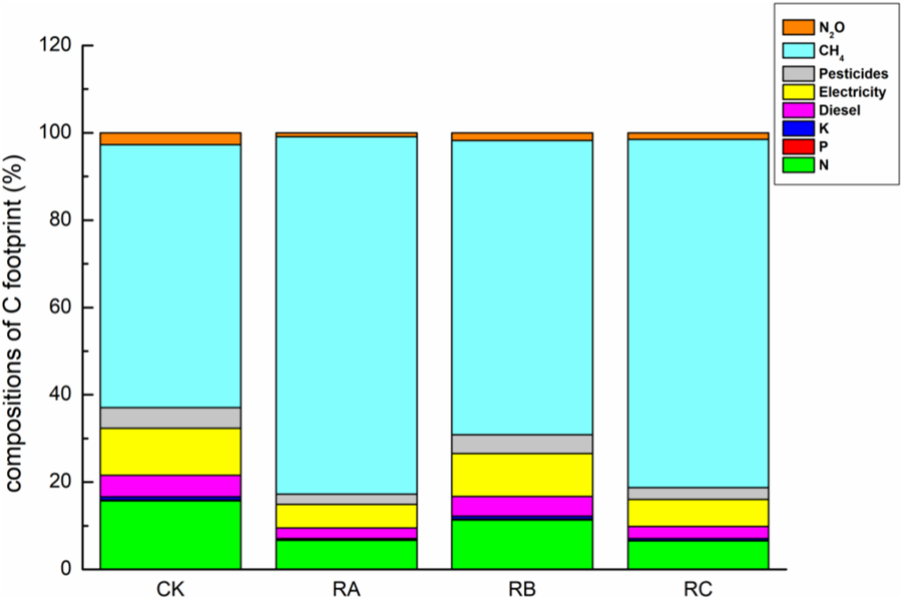
Average annual compositions of C footprint during crop growth season over the 2 years

**Fig. 2:**
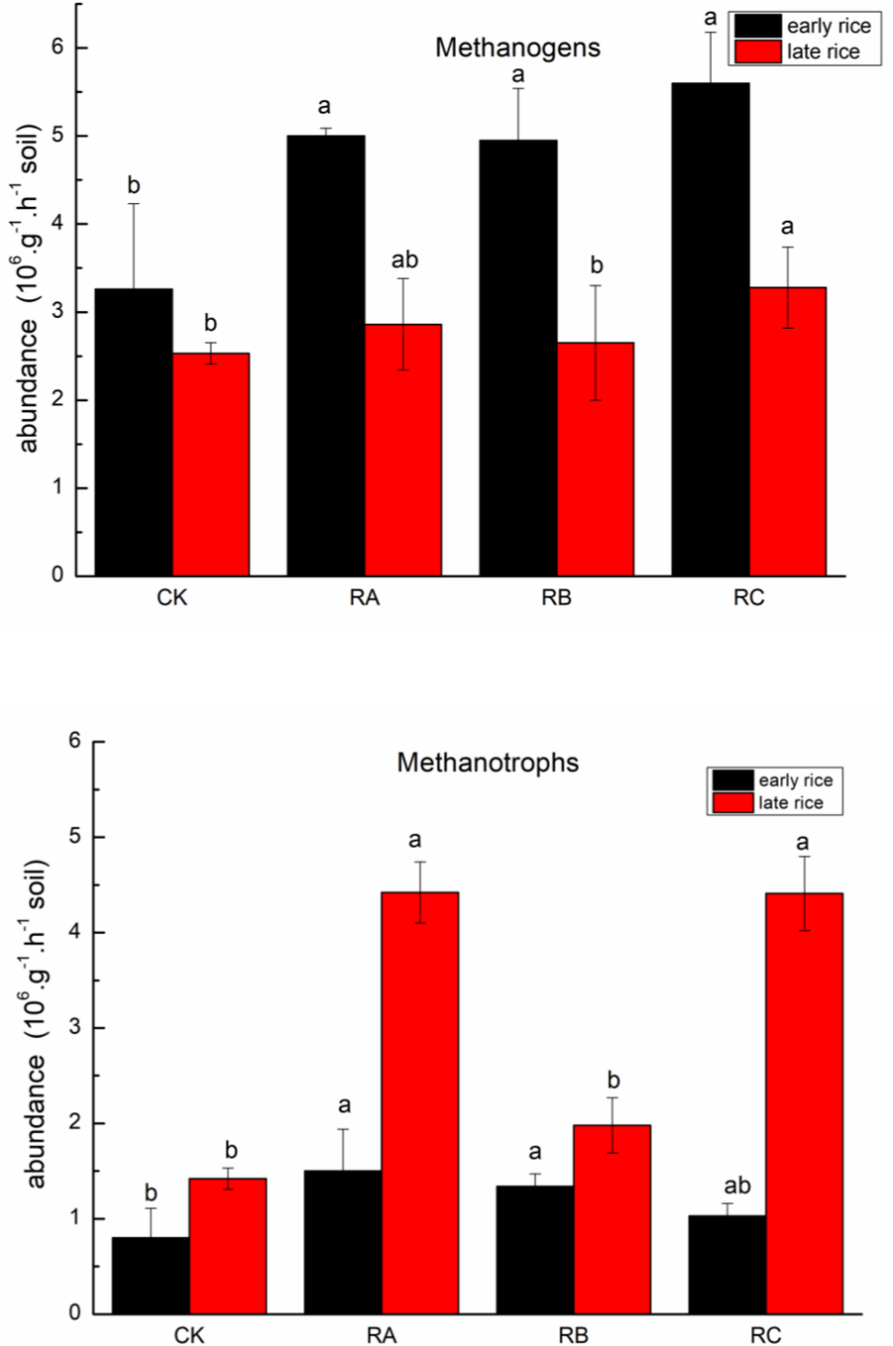
Abundances of methanogens and methanotrophs during the 2018 rice season in response to incorporation of Chinese milk vetch and rice straw combined with reduced chemical fertilizer. Different lowercase letters in the same column indicate significant differences among the treatments at *P* ≤ 0.05

The C emissions per unit area of all the treatments was 9731 to 19,584 kg CO_2_-eq ha^−1^ and the C footprint per unit production was 0.52–1.01 kg CO_2_-eq kg^−1^. The C footprint of all the treatments is mainly from C output of soil CH_4_, N fertilizer and electricity consumption for irrigation (Table 2), accounting for 60.25–81.88%, 6.64–15.73% and 5.35–10.77%, respectively (Fig. 1). Compared with C footprint of control CK, treatments RA and RC increased by 60.32% and 34.92%, while treatment RB decreased by 17.46%, which may attributed to the less N fertilizer application amount, lower C output of CH_4_ and N_2_O as well as higher yield of treatments RB (Table 3). Our result was consistent with previous studies which reported soil CH_4_ was dominate source of C footprint in paddy field [38, 39]. Compared with control CK, treatments RA, RB and RC enhanced CH_4_ emissions, mainly resulting from the following aspects: (1) The continuous flooded irrigation provided a favorable anaerobic environment for the growth and reproduction of methanogens and methanotrophs (Fig. 2) [40–42]; (2) Mulching and retention of rice straw and *Astragalus sinicus* L. could maintain soil moisture, provide organic matter for soil and reduce soil redox potential, thus leading to CH_4_ emissions increase [43, 44]; (3) Organic materials retention supplied methanogenic bacteria with adequate substrates [11, 45, 46], while the decomposition of straw consumed oxygen, enhanced soil anaerobic environment and inhibited the activity of methane oxidizing bacteria, thus promoting CH_4_ emissions [47]; (4) The application of mineral fertilizer and the decomposition of organic materials accelerated the rice and its root growth, thus making the secretion and abscission of rice root increase and providing a substrate for related microorganisms, resulting in the rapid increase of CH_4_ emissions [48].

Fertilizer and study year had significant interactive effect on C footprint (Table 5). Fertilizer (mineral fertilizer combined with organic materials) had different effect on GHG emissions when the rainfall and temperature were different over the 2 years, therefore, there exists an interactive effect between fertilizer and year. Different temperature and rainfall can affect the evaporation and loss rate of N fertilizer, thereby affecting N_2_O emissions because there was a linear relationship between N_2_O emissions and N fertilizer [49, 50]. Meanwhile temperature, rainfall and crop straw retention also affect soil moisture and aeration condition, thus affecting GHG emissions. CH_4_ is produced in an anaerobic environment [51]. Nitrification is sufficient when the soil contains sufficient oxygen, while denitrification mainly occurs in poor oxygen environments in soils [52, 53]. Moreover, rainfall can improve the temperature of soil water, enhance microbial activity, increase organic matter or nitrogen mineralization rate, and promote the rapid release of large amounts of C and N in soil in a short period, thus promoting GHG emissions [54–56].

### NECB

The NECB can be used to assess the short-term net C budget balance via C input and output in an agro-ecosystem [57]. For control CK and the treatments with retention of *Astragalus sinicus* L. and rice straw combined with different amount of reduced mineral fertilizer, C input of crops varied from 31.98 Mg CO_2_-eq ha^−1^ to 35.85 Mg CO_2_-eq ha^−1^ and C output ranged from 26.59 Mg CO_2_-eq ha^−1^ to 40.79 Mg CO_2_-eq ha^−1^. Control CK and treatment RB became C sink compared with treatments RA and RC because control CK was winter fallow and its C output was the least and treatments RB had the most crop biomass and C input (Table 4). Straw retention had significant effect on crop biomass and C input. The effect of study year as well as fertilizer * year on NECB was significant (Table 5).

**Table 4:** Assessment of C budget and balance in different treatments (Mg CO_2_ ha^−1^)

Items	CK		RA		RB		RC	
	C input	C output	C input	C output	C input	C output	C input	C output
C input of Chinese milk vetch and rice	31.98		35.37		35.85		32.70	
GHG (direct and indirect)		9.73		19.58		10.64		17.03
CO_2_ cumulative emissions		16.86		21.21		21.64		19.78
Total	31.98	26.59	35.37	40.79	35.85	32.29	32.70	36.80
NECB	5.39		− 5.42		3.56		− 4.1	

GHG represents greenhouse gas; CO_2_ represents carbon dioxide; C represents carbon; NECB represents net ecosystem carbon budget

**Table 5:** Interactions of straw retention, fertilizer and study year on mean GHG, CO_2_, C input, C footprint, crop biomass and NECB during the crop growing season

	GHG	CO_2_	C input	C footprint	Crop biomass	NECB
Straw retention^a^						
−SR	9731.44	16,860.67	31,981.66	0.65	15,208.83	5.39
+ SR	15,748.89**	20,879.21	34,641.17*	0.79	20,044.44***	− 1.99
Year^b^						
2018	14,235.30	26,169.29	32,901.97	0.78	18,278.67	− 7.50
2019	14,253.75	13,579.86 ***	35,050.62	0.73	19,392.42	7.22***
F-values						
Fertilizer * year	51.458 ***	49.338***	0.924	6.271**	1.000	6.689**

F-values are provided for interactions

GHG represents greenhouse gas; CO_2_ represents carbon dioxide; C represents carbon; NECB represents net ecosystem carbon budget; − SR represents no straw (*Astragalus sinicus* L. and rice) retention; + SR represents straw (*Astragalus sinicus* L. and rice) retention

There were significant interactions (fertilizer * year) for the six variables. *(0.01 < *P* ≤ 0.05), **(0.001 < *P* ≤ 0.01), or ***(P ≤ 0.001) are used to represent significant effects among the treatments

^a,b^Values were averaged across different treatments, crop, and study years

CO_2_ emissions contributed to the largest proportion of C output. CO_2_ emissions was significantly affected by straw retention (Table 5). CO_2_ emissions from treatments RA, RB, and RC were higher than that of control CK (Table 4), which might result from the accumulation of soil total organic carbon, microbial biomass carbon, and dissolved organic carbon caused by *Astragalus sinicus* L. and straw retention. Moreover, the application of mineral fertilizer and the decomposition of straw also promoted the growth and reproduction of soil microorganisms, thus enhancing soil respiration and promoting soil CO_2_ emissions [58–62]. With the growth of *Astragalus sinicus* L. and rice plants, crop root secretion and abscission increased, which strengthened the microbial activity and rice respiration, thus increasing CO_2_ emissions [63, 64]. In addition, straw C decomposition also stimulated the mineralization of SOC to produce CO_2_ [65].

## Conclusion

The GHG emissions of treatments RA, RB, and RC with organic material retention combined with reduced mineral fertilizer at the rate of 15%, 27.5%, and 40% respectively increased by 9.30–101.25% over the two years compared with that of control CK. The increase resulted from increased soil CH_4_ emissions, which occupied more than 60%. Meanwhile treatments RA, RB, and RC increased the yield (including *Astragalus sinicus* L., and rice biomass) by 28.08–34.99% compared with that of control CK. Treatment RB decreased C footprint which mainly attributed to reduced N fertilizer and higher bimass compare with control CK. Treatment RB (*Astragalus sinicus* L. and rice straw retention with reduced N fertilizer by 27.5%) became C sink because increased C input outweighed the increased C output. These results suggest that treatment RB is better in reducing chemical fertilizer amount, increasing crop yield and C input, which is more conductive to sustainable development of agriculture.

## Supplementary Information

**Additional file 1: Figure S1.** Experiment site.

## Data Availability

The data that supports the findings of this study are available in Additional file 1.

## Abbreviations

C: Carbon
N: Nitrogen
CO_2_: Carbon dioxide
GHG: Greenhouse gas
CH_4_: Methane
N_2_O: Nitrous oxide
SOC: Soil organic carbon
NECB: Net ecosystem carbon budget
