# Supplementary figures and images for: Comparison of carbon footprint and net ecosystem carbon budget under organic material retention combined with reduced mineral fertilizer
Source: Carbon Balance Manag. 2021 Mar 1;16:7. doi: 10.1186/s13021-021-00170-x (PMC7923666; doi:10.1186/s13021-021-00170-x)

Additional Figures:


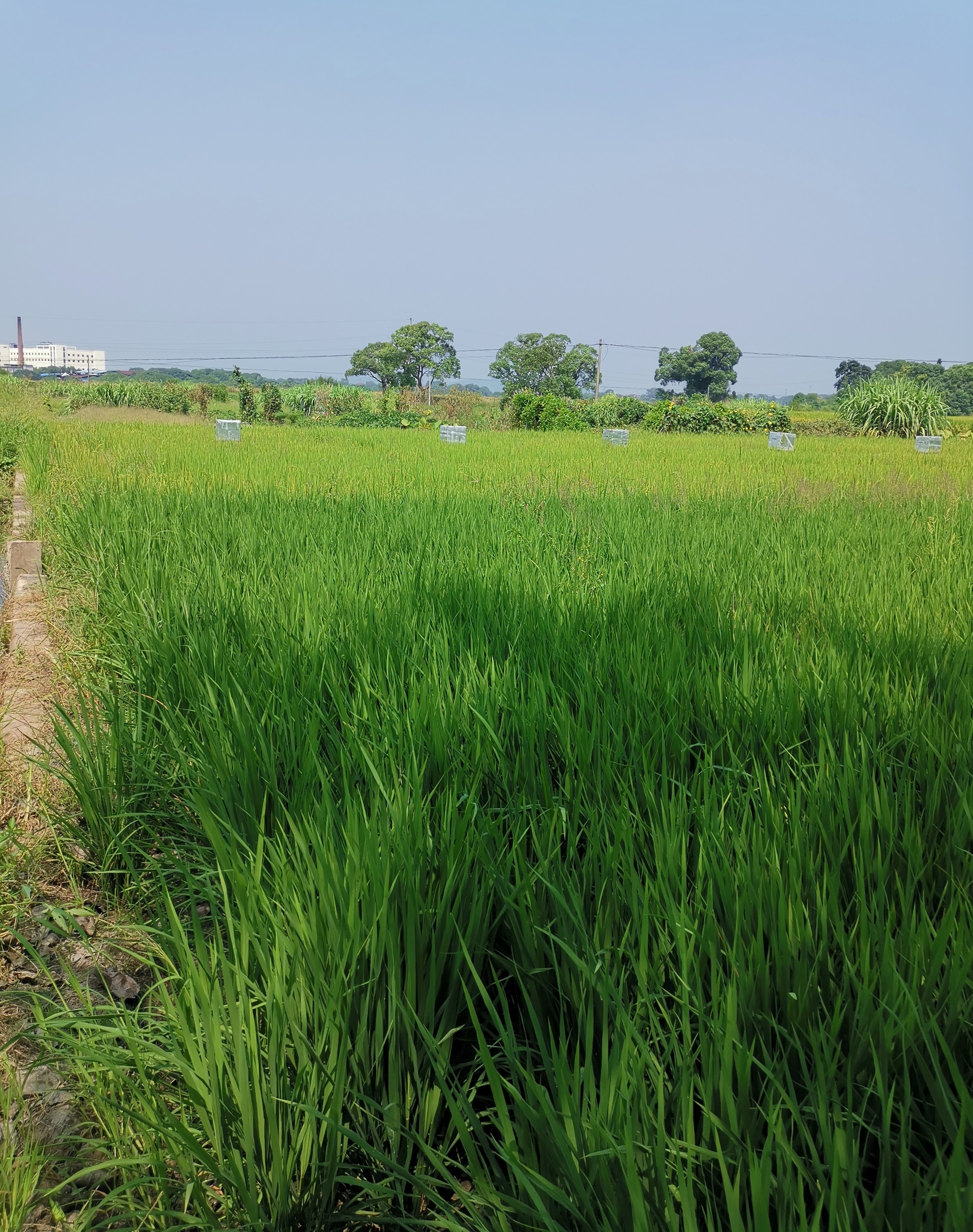


Fig. S1 Experiment site

Supplement: Supplementary file 1 — Additional file 1: Figure S1. Experiment site. [file 13021_2021_170_MOESM1_ESM.doc]
